# Research Progress in Intestinal Microecology in Pancreatic Cancer Diagnosis and Treatment

**DOI:** 10.1155/2022/6069403

**Published:** 2022-12-03

**Authors:** Zetao Han, Haiyan Zhang, Lu Lu, Xin Li, Caoyu Zhang, Jiajie Zhu, Chaonan Li, Qingjing Wang, Keda Chen

**Affiliations:** ^1^Shulan International Medical College, Zhejiang Shuren University, Hangzhou 310000, China; ^2^Zhejiang Shuren College, Zhejiang Chinese Medical University, Hangzhou 310000, China

## Abstract

The intestinal microbiota has an increasingly recognized role in the development of cancer, in which microbial interactions play a more important than expected role. Pancreatic cancer is a highly fatal disease, in which its mortality is closely related to its morbidity. Early detection is the best chance of improving survival. Through an in-depth understanding of the pancreatic cancer microbiota, we could establish screening or early diagnosis methods for pancreatic cancer, implement bacterial treatment, adjust the therapeutic effect, and even reduce adverse reactions. These would lead to new developments and provide hope for patients with pancreatic cancer. Herein, we review the progress in intestinal microbiology research to diagnose and treat pancreatic cancer.

## 1. Introduction

Among the most common malignant tumors of the gastrointestinal tract, pancreatic cancer has an extremely high degree of malignancy and one of the worst prognoses among cancers. The incidence of pancreatic cancer is increasing. The only available treatment is surgical resection; however, only about 20% of patients are suitable for resection at the time of diagnosis, and the rate of surgical mortality is prohibitive. After surgical removal, the average survival is 10–20 months [1]. Pancreatic cancer, with its insidious and typical clinical symptoms, is a gastrointestinal malignancy that is difficult to diagnose and treat. The most common form of pancreatic cancer is pancreatic ductal adenocarcinoma (PDAC) [2, 3]. In recent years, pancreatic cancer's mortality and morbidity rates have increased significantly.

The majority of patients with PDAC present with locally advanced (40%) or metastatic (40%) disease, and standard medical treatment consists primarily of palliative systemic therapy. Despite treatment options such as surgery, chemotherapy, and biotherapy, patients with PDAC at all stages combined have a 10% 5-year survival rate, while for those with distant metastases it is only 3% [4]. Recent studies have demonstrated that intestinal microecology could have an important role in pancreatic cancer development and that bacteria are associated with pancreatic disease pathogenesis, such as PDAC and autoimmune pancreatitis [5–11]. This review examines how intestinal microecology is used to diagnose and treat pancreatic cancer.

## 2. The Role of the Microbiome in the Development and Progression of Pancreatic Cancer

The etiology of pancreatic cancer is unclear, and its specific mechanism is still being studied. Oral bacterial taxa, including *Fusobacterium* and *Granulicatella Adiacens*, are present and enriched in the cystic fluid of intraductal papillary mucinous neoplasms (IPMN) with high atypia. Elevated bacterial DNA and interleukin-1*β* in pancreatic sacs have a synergistic effect on IPMN and tumor grade [12].

**Figure 1:**
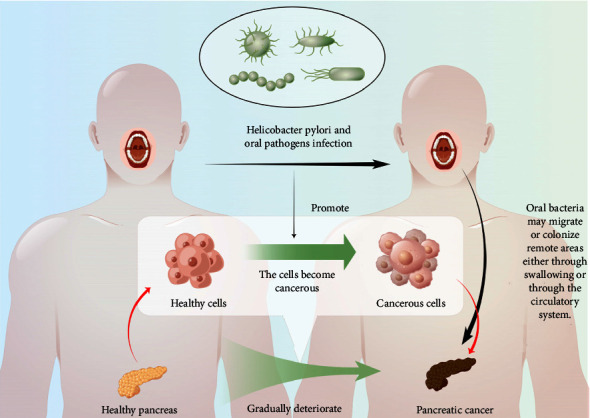
This figure shows that in the development and progression of pancreatic cancer, oral bacteria might migrate or colonize the pancreas through swallowing or through the circulatory system, changing the pancreatic microenvironment, and then induce carcinogenesis of normal pancreatic cells.

The salivary microbiome of patients with pancreatic cancer was analyzed using the human oral microbial identification microarray (HOMIM). The results showed that, with a sensitivity and specificity of 96.4% and 82.1%, respectively, *Neisseria elongata* and *Streptococcus medialis* could distinguish patients with pancreatic cancer from healthy individuals [13]. The study also found that exposure to *Porphyromonas gingivalis*, a pathogen associated with periodontal disease, might lead to an increased risk of pancreatic cancer because of the high levels of anti-*Pseudomonas gingivalis* antibodies found in patients with pancreatic cancer [13]. A sequence analysis of the 16SrRNA gene indicated that the presence of *Pseudomonas gingivalis* and its associated *Actinobacillus* in the oral cavity was associated with the occurrence of pancreatic cancer. Li et al. found that swallowing or through the circulatory system, oral bacteria could migrate and colonize remote areas (Figure 1) [14]. A study examined the tongue coating microbiota of 25 healthy controls and patients with 30 PDAC and found that patients with PDAC patients had a significantly enriched microbiota of their tongue coating, which differed significantly from that of controls. Patients with PDAC could be differentiated from the healthy controls according to their small number of certain bacteria (*Haemophilus*, *Porphyromonas*, *leptothrix*, and *Clostridium*) [15].

Geller et al. showed that there are bacteria in pancreatic cancer tissues, and these bacteria can promote the further deterioration of pancreatic cancer in different ways [16]. Zitvogel et al. showed that pancreatic cancer was associated with a specific intestinal microecological composition and metabolite richness [17]. Cancer-related microorganisms found in the tumor environment of the pancreas might reach the pancreas through ventral intestinal metastasis of the pancreatic duct [18]. These results suggest that pancreatic cancer is related to duodenal microbiological changes, and some factors that regulate the intestinal microflora are closely associated with pancreatic cancer occurrence and development.

A *Helicobacter pylori* infection, along with oral pathogens, such as *Porphyromonas gingivalis, Neisseria longchain, Streptococcus*, etc., contribute to PDAC [19]. In addition, Aykut et al. showed that the fungal microbiota promotes pancreatic cancer [20]. Fungal migration from the intestinal lumen to the pancreas is associated with PDAC pathogenesis. Pancreatic cancer is promoted by the fungal activation of mannose-binding lectin (MBL). A pattern recognition receptor (PRR) from the innate immune system binds polysaccharides in fungal walls to activate the complement cascade [21, 22]. In tumor cells, loss of MBL or complement C3 in the equatorial region and deletion of the complement C3a receptor (C3aR) can prevent tumor progression [20, 23].

Sethi et al. showed that the growth of pancreatic tumors in mice is affected by the gut microbiota by regulating immune responses, and in a mouse pancreatic cancer model, depletion of the gut microbiota by oral antibiotics significantly reduced the tumor burden [24]. By contrast, in Rag1 (encoding recombination activating 1) knockout mice (which lack mature B and T cells), gut microbiome depletion could not prevent tumor development. Depletion of the intestinal microbiota resulted in a significant increase in T cells producing interferon gamma (IFN*γ*) and a corresponding decrease in T cells producing interleukin (IL)-17A and IL-10 [24]. The study also found that reducing the gut microbiota led to pancreatic tumors and effector T-cell infiltration [24].

Pancreatic cystic neoplasms (PCN) are being detected at an increasing rate in the general population. The latest findings suggest that bacterial abundance might be increased in pancreatic PCN tissues compared with those in the normal pancreas [25]. A retrospective study using endoscopic ultrasound fine needle aspiration (EUS-FNA) revealed that regardless of the cyst type or clinical and biochemical parameters, the sac fluid of PCNs contained a unique microbiome [26].

Therefore, the diagnosis and treatment of pancreatic cancer have yet to be explored in association with the significant role played by the microbiome in the occurrence and development of pancreatic cancer.

## 3. Pancreatic Cancer Diagnosis via Intestinal Microbiological Monitoring

A study reported that in excess of 75% of cases of pancreatic cancer are at stage III or IV upon diagnosis and are therefore considered as advanced disease [27]. At present, PDAC has a poor prognosis, and radical surgery is still the only treatment option (surgery followed by chemotherapy) [28]. Early detection of PDAC can improve the quality of life and survival chances of patients. At present, pancreatic cancer diagnosis is based mainly on symptoms, signs, imaging manifestations, tumor markers, histopathology, and/or cytology. However, all this evidence was confirmed after the onset of pancreatic cancer, and positive results are found in the middle and late stages. Early diagnosis of pancreatic cancer is still very difficult with no population-level screening tools or biomarkers [29]. Mendez et al. found that for early pancreatic cancer detection, the altered microbiota could be used as a predictive marker [30]. In the early stages of PDASC, large number of Proteus were found. Moreover, the polyamines and nucleotide biosynthetic pathways have been studied to assess their functional importance in tumor progression. Therefore, changes to the microbiota and the release of metabolites that promote host tumorigenesis are closely linked to early PDAC. However, effective initial diagnosis continues to be difficult, and more specialized biomarkers are needed [31]. A study utilizing HOMIM found that patients with pancreatic cancer could be distinguished from healthy people by the presence of *Neisseria longate* and *Streptococcus mitis*, with a sensitivity and specificity of 96.4% and 82.1%, respectively [13]. The presence of two biomarkers in oral saliva, *Neisseria longate* and *Streptococcus mitis*, can be used as diagnostic tools for the early stage of pancreatic cancer. Torres et al. showed that patients with pancreatic cancer have an increased percentage of *leptotrichia* and a reduced amount of *porphyromonas gingivalis* in their saliva in comparison with healthy subjects, and that ratio of the two (LP ratio) can serve as a biomarker of pancreatic cancer [32]. One study evaluated the intestinal flora and its metabolites in KPC mice (a pancreatic ductal adenocarcinoma (PDA) model) and patients with PDAC for the early detection of PDAC [30]. The study found that early in the development of PDAC, the colonic flora was enriched with *proteobacteria* and *firmicutes*. This was associated with elevated levels of serum polyamines, a product of active metabolic pathways. Thus, the gut microbiota is a potential noninvasive tool for the early detection of PDAC [30, 33].

Studies on the relationship between deregulation of the fungal microbiota and the progress of PDAC are limited. To develop a prognostic tool for PDAC, further studies should also consider fungal profiles. With the rapid advance of medical science, prompt analysis of the intestinal microflora might form the basis for the prevention and early diagnosis of pancreatic cancer.

## 4. Role of Intestinal Microecology in the Treatment of Pancreatic Cancer

### 4.1. Treatment with Probiotic Derivatives

#### 4.1.1. Short Chain Fatty Acid (SCFA) Therapy

**Figure 2:**
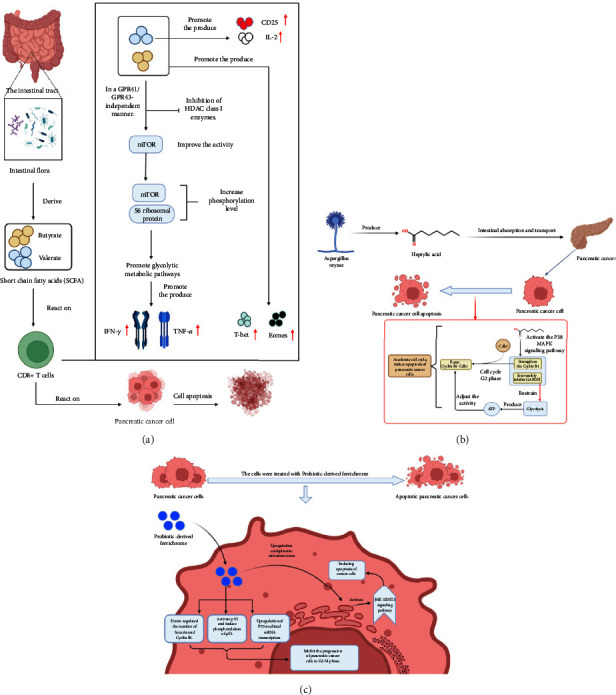
(a) Microbial SCFAs butyrate and valerate modulate the responses of CD8^+^ T cells thus improving adaptive immunotherapy for tumors. (b) The antitumor effect of heptanoic acid produced by *Aspergillus oryzae* on pancreatic cancer. (c) The antitumor effect of high iron pigment derived from probiotics on pancreatic cancer. SCFAs, short chain fatty acids; CD25, interleukin 2 receptor subunit alpha; IL-2, interleukin 2; GPR41, G-protein coupled receptor 41; GPR43, G-protein coupled receptor 43; HDAC, histone deacetylase; mTOR, mechanistic target of rapamycin kinase; IFN*γ*, interferon gamma; TNF*α*, tumor necrosis factor alpha; T-Bet, T-box transcription factor 21; eomes, eomesodermin; CDK1, cyclin dependent kinase 1; MAPK, mitogen activated protein kinase; GAPDH, glyceraldehyde-3-phosphate dehydrogenase; JNK, JUN N-terminal kinase; DDIT3, DNA damage inducible transcript 3.

Butyrate, propionate, and acetate are the primary microbial metabolites, belonging to the SCFA group. They can enhance the expansion of regulatory T cells (Treg cells) and improve the function of effector T cells. In recent years, numerous studies have demonstrated the antipancreatic cancer effects of SCFAs (Figure 2(a)).

Corra et al. found that sodium butyrate can promote the differentiation of human pancreatic cancer cells and induce the expression of certain tumor-associated antigens [34]. Researchers also found that sodium butyrate can inhibit pancreatic cancer cell invasion and metastasis by inhibiting integrity *β*4 [35]. Additionally, butyrate might inhibit the efficiency of anticytotoxic T lymphocyte associated antigen-4 (CTLA-4) immune checkpoint inhibitors (ICIs) by inhibiting the stimulation of tumor-specific memory T cells and T cells by dendritic cells [36]. One study found that valproic acid significantly downregulates pancreatic cancer cell expression of epidermal growth factor receptor (EGFR), ErbB2 receptor tyrosine kinase 2 (ErbB2), and ErbB2 receptor tyrosine kinase 3 (ErbB3) by inducing microRNAs targeting members of the ErbB family. Furthermore, valproic acid's antipancreatic cancer activity was confirmed in a transplanted tumor model [37]. Therefore, valproic acid has selective antitumor activity against pancreatic cancer coexpressing EGFR/ErbB2/ErbB3.

Luu et al. showed that the human symbiotic bacterium *Mobilicoccus massiliensis* is the only bacterium that synthesizes large amounts of the SCFAs, valerate, and butyrate. The valerate and butyrate produced by M. *massiliensis* enhanced CD8^+^ T cell production of effector cytokines [38]. In the presence of valerate, tumor-specific T cells were better able to fight solid tumor models. Luu et al. also found that valerate therapy enhanced the efficacy of chimeric antigen receptor (CAR) T-cell therapy in pancreatic cancer, demonstrating the potential of optimizing CAR T-cell generation by valerate and butyric acid to enhance their efficacy after adoptive transfer [38].

These results suggest that SCFAs inhibit the development, invasion, and occurrence of pancreatic cancer significantly. Thus, regulating the levels of SCFAs through intervention with the colonic flora could positively affect pancreatic cancer prevention and treatment.

#### 4.1.2. Treatment Using Other Derivatives

In addition to SCFAs, other probiotic derivatives can be used to treat pancreatic cancer. Konishi et al. reported that hepatic acid, a cancer suppressor produced by *Aspergillus coryza*, is synthesized in the intestine and transported to other organs, including the pancreas, thereby inhibiting pancreatic cancer growth [39]. The study also showed that hepatic acid enhances Cyclin B1 via the P38 mitogen activated kinase (MAPK) signaling pathway, a key glycolytic enzyme signaling pathway in pancreatic cancer cells. In the G2 phase, the cyclin B1-cyclin dependent kinase 1 (CDK1) complex is formed (the decisive step for progression of the cell cycle into the M phase), which irreversibly inhibits glyceraldehyde-3-phosphate dehydrogenase (GAPDH), thus inhibiting GAPDH-mediated glycolysis to produce ATP. Therefore, regulating the activity of Cyclin B1-CDK1 can accelerate the cell cycle, thus inducing pancreatic cancer cell apoptosis and playing an inhibitory role in cancer (Figure 2(b)).

In addition, Kita et al. showed that probiotic-derived hyperfine pigments inhibit cancer cell progression to the G2-M phase by activating p53 via phosphorylation, which upregulates p53-mediated mRNA transcription and downregulates the amount of secretase inhibitor protein (Securin) and cyclin B1 [40]. At the same time, endoplasmic reticulum stress is upregulated and the JUN N-terminalkinase-DNA damage inducible transcript 3 (JNK-DDIT3) pathway is activated, which promotes the apoptosis of cancer cells and inhibits cancer (Figure 2(c)). Meanwhile, Chen et al. showed that *Lactobacillus* can inhibit pancreatic cancer growth by inhibiting the transforming growth factor beta (TGF-*β*) signaling pathway mediated by *Porphyromonas gingivalis* [41].

### 4.2. Immunotherapy

The intestinal flora promotes immunotherapy against pancreatic cancer by regulating immune checkpoints. As an immunosuppressive molecule an immune checkpoint can inhibit lymphocyte function and allow tumor cells to escape the immune system. The intestinal flora has an important function in the formation of the human immune system and the induction of immune responses. Studies have demonstrated that CTLA-4, as an immune checkpoint inhibitor, is dependent on intestinal flora in the treatment of tumors [42, 43]. When the colonic flora is absent, it cannot produce an effective antitumor effect. By regulating dendritic cell function, intestinal bacteria can regulate the antitumor immune response mediated by T cells. Accordingly, it is beneficial for the prevention and treatment of pancreatic cancer to select an immunotherapy based on altered colonic microbiological conditions.

Studies have demonstrated that a variety of bacteria, including *Akkermansia*, *Fusarium*, *Clostridium*, and nitrobacterium are linked to the antitumor effect of targeted therapy using programmed cell death 1 (PD-1) and programmed cell death 1 ligand 1 (PDL1, also known as the CD274 molecule) [44–46]. Inoculating germ-free mice with selected interferon gamma-induced microbial strains enhanced the efficacy of anti-PD-1 ICIs, and antitumor T-cell responses were significantly promoted [47]. These effects are caused by the influence of microbial metabolites such as butyric acid and propionic acid. In some cases, however, the influences of probiotic compounds show conflicting results. For example, higher SCFA levels in feces were associated with longer progression-free survival or a stronger antitumor response, while higher systemic levels were linked to a poorer treatment response [48].

Other microbial metabolites also affect ICI. For instance, inosine produced by nitrobacterium enhances ICIs by activating A2A receptors on T cells [49]. The direct stimulation of lymph node dendritic cells by *Akkermansia muciniphila* can induce microbial-host interactions in cancer immunotherapy to improve the antitumor effects of ICIs, dependent on IL-12 [46] or the induction of antitumor immune responses by Th1 and CD8^+^ T cells [42, 46].

Thus, it is advantageous to select immunotherapies depending on the intestinal microbiology conditions to prevent and treat pancreatic cancer.

### 4.3. Fecal Microbiome Transplantation Therapies

Fecal microbiome transplantation (FMT) is used to replace a disease-associated microbiota with a healthy configuration. In cancer, the transfer of patient stool samples to ICI-treated sterile or antibiotic-treatedtumor-bearing mice demonstrated that a specific microbiome configuration could drive improved immunotherapy efficacy [50]. Two other studies have shown that FMT administered to germ-freetumor-bearing mice from patients who responded well to ICIs could transfer this ICI reactivity to the recipient mice, while mice receiving an “unresponsive” microbiome did not respond to ICIs [51, 52]. A study demonstrated for the first time that the FMT of a recombinant microbiome in tumor-bearing mice from feces of healthy control patients (HC), short-term survival patients (STS), or long-term survival patients without evidence of disease (LTS-NED) reflected the recruitment or lack of immune cells to the tumor environment in each group, which affected tumor growth [53]. The gut microbiota is causally involved in shaping the immune response to tumors and promoting PDAC progression. The success of these trials were partly determined by the choice of donors and how well the donor material was absorbed by the recipient. Despite these preliminary results, there are several quantifiable, regulatory, and scientific uncertainties that should be addressed before FMT can be adopted routinely, e.g., issues associated with the selection of operative donors and recipients, the preparation of the intestines, and reception procedures. Furthermore, the drivers of the clinical effects of FMT, such as microbial metabolites, phages, or bacteria, are mostly unknown and should be tested in large-scale, prospective clinical trials.

## 5. Current Problems and Future Prospects

Increasing evidence that microbes are linked to the progression and treatment response of pancreatic cancer warrants more comprehensive research to reveal the mechanism by which the microbiota exerts these effects. Microbial targeting strategies could provide new hope for the treatment of pancreatic cancer. The products of the intestinal microbiota, such as SCFAs and certain probiotics, can inhibit the development of pancreatic cancer and enhance treatment efficacy (Table 1); however, this kind of research is in its infancy, and most research is still in the preclinical phase. At present, there is no progress in the relevant research on how to distinguish the abnormal bacterial community associated with pancreatic cancer from the normal symbiotic bacterial community by using antibiotics. Currently, the research progress in this field is temporarily confined to the mouse pancreatic cancer model experiment, and the development of pancreatic cancer can be observed by using broad-spectrum antibiotics to eliminate various intestinal flora. No experiments have been conducted to eliminate one or more types of intestinal flora alone.

Patients with pancreatic cancer are generally in poor physical condition, and most patients will undergo chemotherapy, which is associated with its own burden for patients [54, 55]. For example, chemotherapy can cause digestive reactions, including diarrhea and vomiting, together with damage to the kidneys and liver. The most frequent reaction is a digestive reaction, suggesting that chemotherapy might affect the intestinal flora. In mice with pancreatic cancer transplanted tumors, cimetidine treatment altered the colonic microbial composition [56]. The results revealed a significant reduction in Gram-positive bacteria (39% to 17%) and Gram-negative bacteria (38% to 17%) in the intestines of mice with tumors compared with those in the control group. The proportion of *Proteobacteria* (*Escherichia coli* and *Aeromonas hydrophila*) and *Akermania muciniphila* increased significantly. Control mice with tumors had predominantly Gram-positive and Gram-negative bacteria in the intestinal tract, in which Proteus and vermiform bacteria occupied subordinate positions [57–59]. Corty et al. [60] showed that the use of antibiotics in cancer treatment increased the risk of adverse events, such as hematological and gastrointestinal events. Moreover, Vétizou et al. [42] found that in mouse models of sarcoma, melanoma, and colon cancer, CTLA-4 therapy was rendered ineffective when ampicillin, colistin, and streptomycin were administered together. It was also reported that cyclophosphamide (CTX) treatment did not activate antitumor immunity in mice treated with vancomycin (targeting Gram-positive bacteria), leading to treatment failure [61]. However, Geller et al. showed that in models of colorectal cancer, cimetidine and ciprofloxacin could effectively eliminate bacteria-induced chemotherapy resistance, thereby boosting its effectiveness [16]. Meanwhile, Chen et al. showed that the probiotic *Lactobacillus* and gemcitabine synergistically inhibited tumor growth in a transgenic mouse model of pancreatic cancer [62]. Another study [63] observed that adding antibiotics to chemotherapy could improve its efficacy. An analysis of 169 patients with advanced cancer (including pancreatic cancer) treated with cimetidine was conducted retrospectively, dividing the patients into two groups: a no antibiotics group (treated with solutions containing gramicidin but not antibiotics) and an antibiotic treatment group (using the solution containing cimetidine plus antibiotic treatment). Both groups were assessed for efficacy, overall survival (OS), and progression-free survival (PFS). The results showed that the median PFS and OS metrics were higher in the antibiotic treatment group than in the no antibiotics group. These findings suggested that regulating intestinal microecology or using probiotics in combination with chemotherapy could help to treat pancreatic cancer. Above all, gut microbes can become a pancreatic cancer diagnosis standard, and by adjusting the intestinal microecology, could become a new paradigm for disease treatment. Intestinal microbial products (e.g., SCFAs), combined with immune therapy and FMT could be used to treat pancreatic cancer, giving new hope to patients. Chemotherapy can affect the intestinal microecology, and the use of probiotics and antibiotics with combination chemotherapy in the treatment of pancreatic cancer has excellent potential [64]. However, these therapies might have as-yet-unknown side effects and adverse reactions, which will be a problem requiring further research. More studies are also required to optimize combination therapies to bring better results and improve the prognosis of patients with pancreatic cancer.

## 6. Conclusions

Pancreatic cancer is one of the deadliest malignant tumors. It suffers from poor early diagnosis and a lack of effective treatments. Intestinal microbiology has a vital function in multiple physiological activities. There is a close relationship between pancreatic cancer and the intestinal microecology. With the development of technology and science, the correlation between them will be revealed in detail. Although many studies have focused on microbes and how they contribute to pancreatic cancer occurrence and development, only a few studies have discussed how microorganisms influence pancreatic cancer treatment. In this review, we provide fresh insights into the interactions between the intestinal microbiota and pancreatic cancer and summarize some meaningful perspectives and recommendations for developing innovative treatment approaches and models. The intestinal flora are a significant and complex system, and their regulatory mechanism in pancreatic cancer forms a rich network chain. To date, there has been progress in the early diagnosis of pancreatic cancer; however, we lack additional and effective biomarkers. Intestinal microbiological regulation is a novel concept in disease treatment and offers new hope for patients suffering from pancreatic cancer via products of intestinal flora (e.g., SCFAs), combined immunotherapy, and fecal bacteria transplantation. Moreover, the majority of the studies discussed were conducted using mouse models. Thus, the results should be interpreted with caution because there might be different effects in human applications. Limitations in this field, such as the impact of sample sizes in human clinical studies, the homogeneity of disease stages when considering case studies, and whether there is a clinical correlation between the microbiome and the ethnicity of patients and control populations, must also be addressed. Therefore, more epidemiological studies, basic experiments, and clinical trials are needed to explore these aspects and strive for rapid application in clinical treatment. Pancreatic cancer can be treated by regulating intestinal microecology and future treatments and drug development will increasingly rely on microorganisms.

## Figures and Tables

**Table 1:** Intestinal microecology therapies targeting pancreatic cancer.

Treatment	Classification	Action principle
Probiotic derivative therapy	Sodium butyrate	The expression of integrin *β*4 is downregulated to inhibit the invasion and metastasis of pancreatic cancer cells
	Valproic acid	The expression of EGFR, ErbB2, and ErbB3 in pancreatic cancer cells was significantly downregulated
	Butyric acid	Enhanced effector cytokine production in CD8^+^ T cells; enhanced CAR T cell therapy.
	Pentanoic acid	
	Heptanoic acid	Regulating P38 MAPK signaling pathway (Figure 2(b))
	High-speed pigment	p53-mediated mRNA transcription (Figure 2(c))

Immunotherapy		To improve the antitumor effect of PD-1/L1 targeted therapy
Fecal microbiome transplantation therapy		Fecal microbiota transplantation (FMT) replaces disease-related microbiota with a healthy configuration to improve the intestinal microecology of patients

## Data Availability

The data supporting this systematic review are from previously reported studies and datasets, which have been cited. The processed data are available from the corresponding author upon request.
